# Investigation of Citrinin and Pigment Biosynthesis Mechanisms in *Monascus purpureus* by Transcriptomic Analysis

**DOI:** 10.3389/fmicb.2018.01374

**Published:** 2018-06-28

**Authors:** Bin Liang, Xin-Jun Du, Ping Li, Chan-Chan Sun, Shuo Wang

**Affiliations:** ^1^Key Laboratory of Food Nutrition and Safety, Tianjin University of Science and Technology, Ministry of Education, Tianjin, China; ^2^Beijing Advanced Innovation Center for Food Nutrition and Human Health, Beijing Technology and Business University, Beijing, China

**Keywords:** pigment, citrinin, transcriptomic analysis, *Monascus purpureus*, solid-state fermentation

## Abstract

*Monascus purpureus* YY-1 is widely used in food colorant production in China. Our previous study clearly illustrated the whole-genome data for YY-1 and provided useful insight into evolutionary research and industrial applications. However, the presence of citrinin, which has nephrotoxic, hepatotoxic, and carcinogenic activities, has attracted attention to the safety of *Monascus* products. In an effort to reduce the harmful effects of citrinin in *Monascus-*related products, a random mutant of *M. purpureus* YY-1 with low citrinin production (designated as “winter”) was obtained in this study. To analyze the biosynthesis and regulation mechanisms of pigment and citrinin, a transcriptomic analysis of the *M. purpureus* YY-1 and winter strains was performed. Comparative transcriptomic analysis reveals *pksCT*, the essential gene for citrinin synthesis, showed a low expression level in *M. purpureus* YY-1 and winter, which suggested there might be isoenzymes in *M. purpureus* YY-1 that were responsible for the citrinin synthesis during evolution. In addition, changes in transcription factor expression may also influence the network regulating the citrinin synthesis pathway in *M. purpureus*. Moreover, the yields of pigments produced by the winter mutant were significantly increased. Repressing the central carbon metabolism and improving the acetyl-CoA pool can contribute to a high pigment yield, and enhanced NADPH regeneration can also lead to the metabolic flux of pigment production in *M. purpureus*. Investigations into the biosynthesis and regulation of citrinin and pigment production in *M. purpureus* will enhance our knowledge of the mechanisms behind the biosynthesis of fungal secondary metabolites.

## Introduction

The filamentous fungus *Monascus* has a long history of being used to produce fermented foods in eastern Asia (Shi and Pan, 2011). The health benefits of *Monascus*-fermented products have been described in the Compendium of Materia Medica. *Monascus* species can produce many worthwhile secondary metabolites, such as *Monascus* pigments and monacolin K (Endo, 1979; Feng et al., 2012).

*Monascus* pigments, which are polyketide components, range in structure from tetraketides to octaketides. Representative classes include the anthraquinones, naphthoquinones, hydroxyanthraquinones, and azaphilone structures, each of which exhibits an array of color hues (Mapari et al., 2010). *Monascus* pigments have been used as natural food colorants for over 1000 years worldwide, especially in China. Recently, an increasing number of investigations have shown that *Monascus* pigments exhibit biological activities, such as anti-inflammatory, anticancer and antihyperlipidemic activities (Hong et al., 2008; Lin et al., 2011). In addition, monacolin K is considered an effective agent for reducing blood cholesterol levels (Su et al., 2003). However, *Monascus spp*. also produce citrinin, a toxic product that has nephrotoxic and hepatotoxic effects in animals and humans (Blanc et al., 1995). Many countries, especially Japan, European countries and the United States, have enacted legislation to limit the content of citrinin in *Monascus*-fermented products (Endo, 1979; Blanc et al., 1995). Thus, citrinin contamination has become a hindrance to the export of *Monascus*-fermented products from China.

The generation of non-citrinin-producing strains for use in the commercial production of *Monascus*-related products is a primary task. Although the optimization of culture conditions is a traditional strategy for decreasing the content of citrinin, it is hard to block citrinin biosynthesis completely in *Monascus*. Moreover, random mutagenesis and screening is widely used in the development of low- or non-citrinin-producing mutants, but some mutants are not genetically stable (Shao et al., 2014). Therefore, it is necessary and urgent to clearly understand the biosynthetic pathways involved in *Monascus*-fermented products.

In recent years, several genes related to the biosynthesis of citrinin and pigments have been cloned and characterized. Shimizu et al. first cloned a polyketide synthase (PKS) gene from *Monascus purpureus*. The *pksCT* disruptant lost its ability to produce citrinin, suggesting that *pksCT* plays a critical role in citrinin biosynthesis. Unfortunately, the *pksCT* mutant was not genetically stable, and citrinin production was restored after successive cultivation (Shimizu et al., 2005). Similarly, deletion of *pksCT* in *Monascus aurantiacus* resulted in dramatically decreased citrinin production. Intriguingly, this mutant exhibited a stronger ability to produce red and yellow pigments (Fu et al., 2007). In addition, fungal secondary metabolite pathways are also tightly controlled at the transcriptional level. Shimizu et al. have identified a major transcriptional activator (CtnA) of citrinin biosynthesis in *M. purpureus*. The deletion of *ctnA* significantly decreased the production of the *pksCT* transcript, leading to reduced citrinin production (Shimizu et al., 2007).

Compared with the citrinin biosynthesis pathway, the mechanism regulating the biosynthesis of *Monascus* pigments is more complicated. The first pigment biosynthetic gene cluster was obtained by T-DNA random mutagenesis in *M. purpureus*. The transcriptional regulator gene *mppR1* and PKS gene *MpPKS5* are two major components of pigment biosynthesis (Balakrishnan et al., 2013). Chen et al. systematically investigated the pigment gene cluster in *Monascus ruber* M7. The wild-type strain M7 could produce red, orange, and yellow pigments. However, the *MpigE* disruption strain could only produce four kinds of yellow pigments and could barely produce red pigments. Intriguingly, the mutant could restore pigment production by supplementation with M7PKS-1, an intermediate in the production of *Monascus* pigments (Liu et al., 2016). Furthermore, the overexpression of *MpigE* had positive effects on pigment formation and led to a decrease in citrinin production (Liu et al., 2014). These researchers also identified and characterized a pigment regulatory gene (*pigR*). The *pigR* deletion strain could no longer produce pigments, but its capacity to produce citrinin was greatly enhanced (Xie et al., 2013). These results suggested a close relationship between the pigment and citrinin biosynthesis pathways.

*Monascus purpureus* YY-1 is widely used in food colorant production in China. Our previous study clearly illustrated the whole-genome data for YY-1 and provided useful insight into evolutionary research and industrial applications (Yang et al., 2015). In this paper, a random mutant with low citrinin production was obtained through protoplast transformation. We performed comparative transcriptomic analysis between the wild-type strain YY-1 and the mutant, which revealed the mechanisms underlying pigment and citrinin biosynthesis. Further investigations into the mechanisms regulating pigment and citrinin biosynthesis are necessary. The scientific elucidation of the complex relationship between pigment biosynthesis and citrinin biosynthesis in *Monascus* will broaden our knowledge of the mechanisms involved in the biosynthesis of fungal secondary metabolites and will provide important insights into the genetic engineering of industrial strains to increase the production of specific metabolites.

## Materials and Methods

### Strain and Culture Conditions

*Monascus purpureus* YY-1 was obtained from Fujian Province in China (Yang et al., 2015). *Escherichia coli* DH5α was employed for DNA manipulation.

*Monascus purpureus* strains were grown on potato dextrose agar (PDA) at 30°C for 10 days. Spore suspensions were prepared as previously described (Yang et al., 2015). Rice (45 g) was soaked in distilled water overnight and then transferred to a 250 mL Erlenmeyer flask. Subsequently, the rice was autoclaved at 121°C for 20 min. After cooling, the steamed rice was inoculated with a 10% spore suspension and cultivated at 30°C. After 10 days of cultivation, the red yeast rice was dried at 60°C for 12 h and then ground.

### Construction of Random Mutants of *M. purpureus* YY-1 With Deficient Citrinin Secretion

Random mutants were generated by transforming the *M. purpureus* YY-1 strain with a hygromycin B resistance cassette from the plasmid pHPH. *M. purpureus* spores were obtained from the PDA plates and were inoculated in 100 mL spore medium for 40 h at 30°C and 170 rpm. The mycelium was harvested by filtering, washed with lysis buffer twice and then digested for 4 h under shaking at 100 rpm and 30°C. The protoplasts were separated by filtering through three layers of Miracloth and were collected by centrifugation at 1000 g at 10°C for 20 min. Then, the protoplasts were washed twice with 10 mL STC buffer (0.6 M sorbitol, 10 mM Tris-HCl, 10 mM CaCl_2_, pH 6.5). The pellet was resuspended in 400 μL STC buffer, and 10 μg DNA was added to the protoplast suspension, followed by incubation on ice for 20 min. Subsequently, 100 μL PEG800 was added to the suspension, and the suspension was incubated for 5 min at room temperature. The suspension was diluted by adding 4 mL 1 M sorbitol, and centrifuging at 3000 rpm for 10 min. The pellet was resuspended in 2 mL 1 M sorbitol. Two hundred microliters of the suspension was plated onto regeneration medium. The cultures were incubated at 30°C for 12 h and then overlaid with 10 mL regeneration medium containing 100 μg/mL hygromycin B. After 7 days of cultivation, the transformants were picked onto selective medium.

### Quantitative Analysis of Citrinin and Pigments

The red yeast rice powder was extracted with 75% ethanol by ultrasonic extraction for 20 min. After keeping the solution still for 30 min, the concentration of citrinin was determined by high-performance liquid chromatography (HPLC) using an RF-10Axl fluorescence detector (λ_ex_ = 331 nm, λ_em_ = 500 nm) and a ZORBAXE Eclipse XDB C18 column (5 μL, 250 × 4.6 mm). Elution was performed at 38°C with 45% acetonitrile (v/v, pH 2.5) at a flow rate of 1.0 mL/min.

Extraction of pigments for analysis followed a similar protocol to that for citrinin extraction. The red yeast rice powder was extracted with 70% ethanol at 60°C for 1 h. After filtration, the filtrate was measured at 410, 465, and 505 nm.

### RNA Extraction

Mycelia from the *M. purpureus* YY-1 and winter strains for RNA isolation were harvested after 8 days of cultivation. Then, the mycelia were immediately homogenized in liquid nitrogen and stored at -80°C until used for RNA extraction.

Total RNA was extracted using a modification of a method described previously (Tian et al., 2009). The frozen mycelia were ground to a powder in a prechilled mortar with a prechilled pestle and then processed with the RNeasy^®^ Plant Mini Kit (QIAGEN Translational Medicine Company Limited, Germany) following the manufacturer’s protocol. The quantity and concentration of the final RNA samples were measured using agarose gel electrophoresis and the Eppendorf BioPhotometer^®^ basic and stored at -80°C.

### Transcriptome Construction and Analysis

Total RNA samples from each strain were purified with the Qiagen RNeasy Mini kit plus on-column treatment with DNase I to eliminate genomic DNA contamination. Reverse transcription was performed with the PrimeScript^TM^ RT Reagent Kit (Takara Biotechnology Company Limited, Japan), according to the manufacturer’s protocol. The cDNA library was sequenced at Novogene Corporation (Tianjin, China) by means of the Illumina HiSeq 2500 platform. All data in the present study were generated by sequencing independent biological triplicates.

Prior to analyzing the data, the quality of the raw sequencing reads was checked by the FastQC tool (v0.10.0).^1^ Low-quality and dirty reads with adapter sequences and reads with more than 20% of bases with a QA of <25 or with “N” bases were removed using the NGS QC Toolkit (v2.3) (Patel and Jain, 2012). All clean reads were mapped against predicted transcripts from the *M. purpureus* YY-1 genomic sequence (NCBI: samn08978766) using TopHat (v2.1.1) (Trapnell et al., 2012) with at most two mismatches. HTSeq (v0.7.2) (Anders et al., 2015) was employed to calculate the raw counts of reads mapped to unique exons, and the normalized transcript abundance was described by RPKM (reads per kilobase per million mapped reads). Differential gene expression analysis was carried out by DESeq (v1.30.0) (Anders and Huber, 2010), with the raw read count and exon length as inputs. A *P-adj* value <0.05 indicated significantly different expression levels between the YY-1 and winter strains (**Supplementary Table S1**). The raw RNA-seq data are accessible in the Gene Expression Omnibus under accession number GSE107628.

### Quantitative Real-Time PCR

The CFX96 real-time PCR detection system (Bio-Rad, Hercules, United States) was used for quantitative PCR analysis. Reagents were obtained from TOYOBO (One-step qPCR Kit, OSAKA, Japan). The PCR reaction mixture (20 μl) included 75 ng template RNA, 0.4 μM primers and 10 μL RNA-direct SYBR^®^ Green Realtime PCR Master Mix, according to the manufacturer’s instruction. Three replicates were analyzed. The relative transcript level of each gene was calculated by the 2^-ΔΔ*C*_t_^ method, with the expression in the WT as the control and the expression of GAPDH as the internal standard. The primers used in this study are listed in **Supplementary Table S7**.

### Statistical Significance Tests and Data Plotting

Unless otherwise noted, all statistical significance tests were done with a one-tailed homoscedastic (equal variance) *t*-test. The *R* packages used for data plotting included Pheatmap (version 0.7.7) and ggplot2 (version 0.9.3.1).

## Results and Discussion

### Screening and Characterization of the Mutant With Deficient Citrinin Secretion but Hyperproduction of Pigment

**FIGURE 1:**
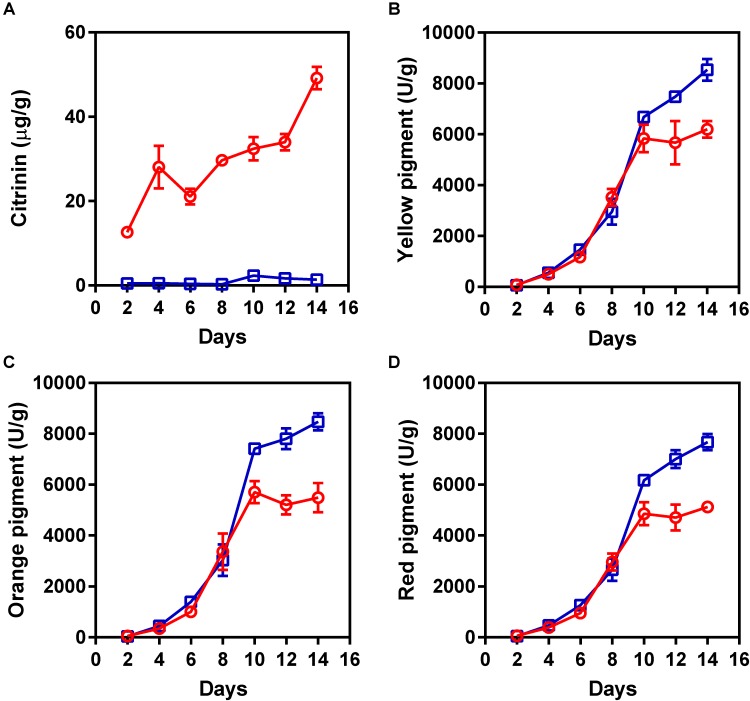
The production of pigments and citrinin by the *Monascus purpureus* YY-1 and winter strains. **(A)** Citrinin, **(B)** yellow pigment, **(C)** orange pigment, and **(D)** red pigment. The red and blue lines represent the YY-1 and winter strains, respectively. The error bars indicate the standard deviations of three independent experiments.

To obtain a non-citrinin-producing mutant, the hygromycin phosphotransferase-encoding gene was introduced into *M. purpureus* YY-1 using protoplast transformation with pHPH, as described previously (Das et al., 1989). A series of hygromycin-resistant insertional mutants of *M. purpureus* was generated and screened for the loss of the ability to produce citrinin. Among these transformants, one transformant showed low citrinin production in solid-state fermentation, and we designated this mutant as “winter.” As shown in **Figure 1A**, citrinin production was significantly decreased in the *M. purpureus* winter strain. After 14 days of cultivation, the citrinin production by the mutant decreased by 35.6-fold, to 1.38 ± 0.22 μg/g, compared to that of the wild-type strain, which produced 49.17 ± 2.64 μg/g of citrinin. Surprisingly, the mutant produced significantly more pigment than the YY-1 strain during the late fermentation period. As shown in **Figures 1B–D**, from the eighth day to the fourteenth day, the pigment production increased significantly. After 14 days of cultivation, the yields of yellow, orange, and red pigment produced by the winter strain reached 8539 ± 426, 8470 ± 332, and 7667 ± 321 U/g, respectively. Compared with the YY-1 wild-type strain, the pigment yields of the winter strain increased by 37% (yellow), 54% (orange), and 49% (red). Thus, the generation of the winter mutant, which exhibited a high yield of pigments and a low yield of citrinin, would greatly reduce the risk of *Monascus*-related products and would provide a foundation for research on the molecular mechanism of citrinin and pigment biosynthesis.

### Transcriptomic Analysis of the *M. purpureus* Winter Strain During Pigment and Citrinin Production

**FIGURE 2:**
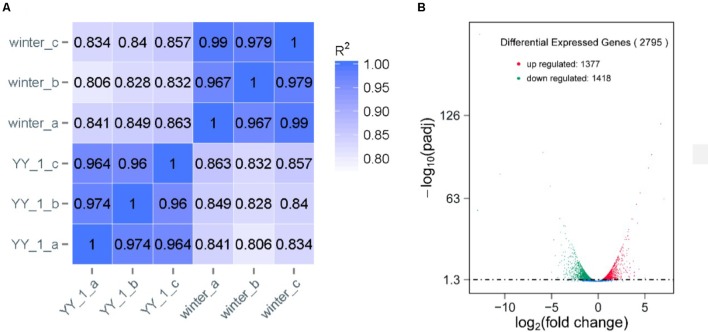
Transcriptomic profiles of the *M. purpureus* YY-1 and winter strains during pigment production. **(A)** Correlation comparison between biological replicates. The Spearman correlation coefficients (*R*^2^) were calculated. **(B)** Differential gene expression. In the statistical analysis of differential gene expression, the transcriptomes derived from the YY-1 strain were used as controls.

To analyze the molecular mechanism of pigment and citrinin production, a transcriptomic analysis of the *M. purpureus* winter strain during pigment production was performed. Due to the rapid increase in pigment production after 8 days of cultivation, total RNA from the mycelia of the *M. purpureus* YY-1 and winter strains was sequenced in independent biological triplicates. The reads per kilobase of exon model per million mapped reads (RPKMs) were calculated as the normalized expression values of each annotated gene. Differential expression analysis was conducted using DESeq (Anders and Huber, 2010). As shown in **Figure 2A**, a high reproducibility of the RNA-seq data from three biological triplicates was observed for the YY-1 and winter strains; the Spearman correlation coefficient was greater than 0.95 for the biological replicates and was less than 0.9 between the different strains (**Figure 2A**). Moreover, the good agreement with the qPCR results demonstrated the reliability of the RNA-seq data (**Supplementary Figure S1**). To discover genes that were significantly upregulated or downregulated between the conditions tested, only genes with a *P*-value from the DESeq analysis of less than 0.001 were analyzed further (**Supplementary Table S1**).

**FIGURE 3:**
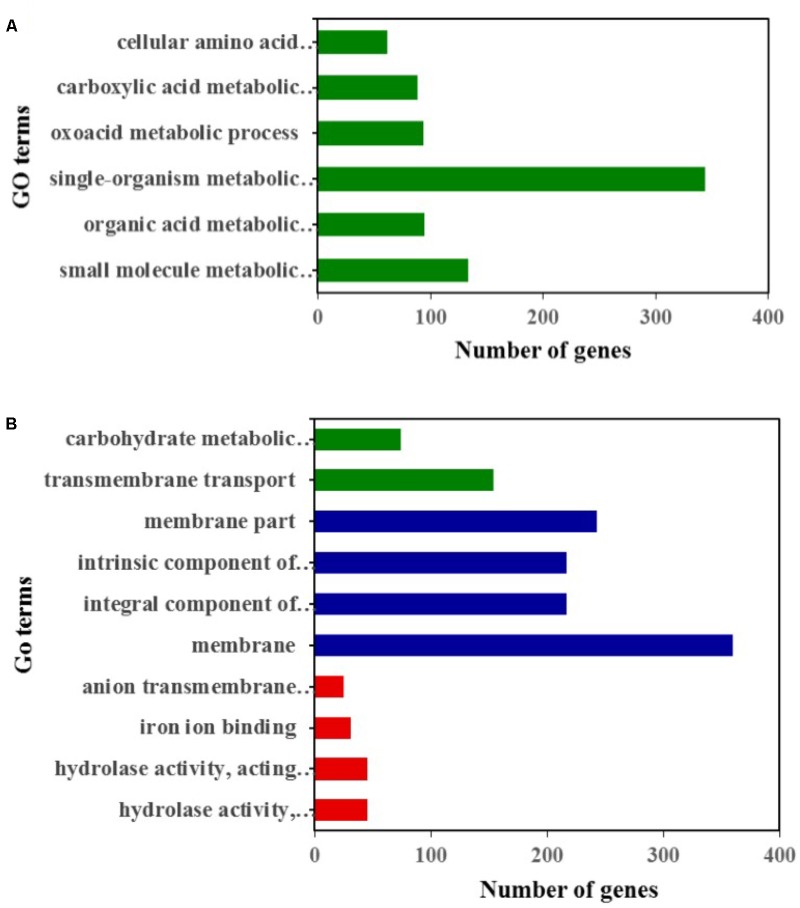
GO classification analysis of genes with significantly upregulated **(A)** and downregulated **(B)** expression levels in the winter strain compared to those in the YY-1 strain. Green, blue, and red represent biological processes, cellular components, and molecular functions, respectively.

Compared to the YY-1 strain, 1377 genes in the winter strain showed significantly increased expression levels (**Figure 2B** and **Supplementary Table S1**). Gene Ontology is a standardized system for gene functional classification, which includes biological process, cellular components and molecular functions of gene products (Chicco and Masseroli, 2016). To further elucidate the types of genes with significantly different expression levels, GO enrichment analyses were performed. The result showed that the genes with increased transcription levels in the winter strain were enriched in several biological processes at a *P*-value of less than 0.05, including the small molecule metabolic process, organic acid metabolic process, single-organism metabolic process, oxoacid metabolic process, carboxylic acid metabolic process, and cellular amino acid metabolic process (**Figure 3A** and **Supplementary Table S2**). The oxoacid metabolic process is related to the metabolism of ketone compounds, including pigments and citrinin. Several genes clustered in the functional category of organic acid metabolic processes and involved in two potential pathways for acetyl-CoA biosynthesis, which is a precursor for pigment biosynthesis, showed upregulated expression levels in the winter strain.

In the Gene Ontology analysis of 1418 genes with significantly decreased expression levels, we found that the downregulated genes were involved in three functional classifications, including the biological process, cellular components and molecular functions of gene products. In the biological process enrichment analysis, transmembrane transport and the carbohydrate metabolic process were significantly enriched. In the carbohydrate metabolic process cluster, the transcription levels of 26 genes involved in carbohydrate degradation were significantly decreased (**Supplementary Table S4**), which might result from a reduction in biomass accumulation when secondary metabolites are synthesized.

Thus, a large percentage of genes showing decreased expression levels participated in metabolic- and transporter-related roles. In the cellular component enrichment analysis, the genes enriched were mainly involved in membrane structure. The molecular function enrichment analysis function showed that hydrolase activity, iron ion binding, and anion transmembrane transporter activity were significantly enriched (**Figure 3B** and **Supplementary Table S3**).

### Regulation of Citrinin Biosynthesis in *M. purpureus*

**FIGURE 4:**
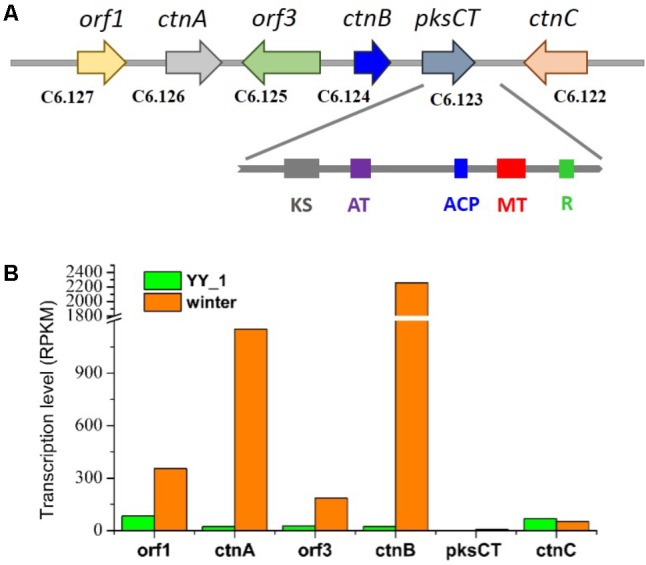
Gene cluster for citrinin synthesis **(A)** and transcription levels **(B)**. KS, ketosynthase; AT, acyltransferase; ACP, acyl carrier protein; MT, methyltransferase; R, reductase.

Citrinin, a mycotoxin which has nephrotoxic activity in mammals, was isolated from most cultures of *Monascus* strains in 1993 (Endo, 1979). Citrinin targets the kidney, resulting in teratogenicity, carcinogenicity, and mutagenicity. Therefore, decreasing the content of citrinin is becoming a necessity that should be addressed as soon as possible. This study included a comprehensive transcriptomic analysis to elucidate the citrinin synthesis pathway in *M. purpureus*. The citrinin synthesis gene cluster comprised *pksCT*, *ctnA*, *orf1*, *orf3*, *ctnB*, and *ctnC*. According to previous reports, *pksCT*, which encodes a multifunction protein that contains putative domains for ketosynthase, acyltransferase (AT), acyl carrier protein (ACP), and a rare methyltransferase, was identified by gene disruption as the key factor for citrinin synthesis in *Monascus aurantiacus* and *Monascus purpureus* (Shimizu et al., 2005; Fu et al., 2007). Surprisingly, in spite of the high expression level of the activator *ctnA* gene, *pksCT* exhibited an extremely low expression level; the RPKM values were 6.45 and 2.01 in the *M. purpureus* winter and wild-type strain, respectively (**Figure 4**). This phenomenon was not consistent with the high production of citrinin in the *M. purpureus* YY-1 strain. The result suggested that *pksCT* may not be the only PKS gene involved in citrinin synthesis. To some extent, this possibility was suggested by the study performed by Shimizu et al. (2005). They found that a *pksCT* disruptant was unstable and could revert its phenotype with repeat cultivation. We speculated that *M. purpureus* YY-1 might have evolved isoenzymes of *pksCT*, such as C2.25 and C6.140, which are responsible for citrinin synthesis; the transcription levels of these purported isoenzymes changed by 2.2- and 2.6-fold, respectively, in the *M. purpureus* winter strain.

The expression levels of *orf1*, *ctnA*, *orf3*, and *ctnB* were upregulated in the winter strain relative to that in YY-1 strain, especially *ctnA* and *ctnB*, which exhibited 50- and 98-fold changes, respectively, although inefficient citrinin synthesis was detected in the winter strain (**Figure 4**). This observation is inconsistent with the report that CtnA, which contains a typical Zn(II)2Cys6 DNA binding motif, functions as an activator of *pksCT* and *orf5* and that its disruption leads to the reduction of citrinin production to barely detectable levels (Shimizu et al., 2007).

**FIGURE 5:**
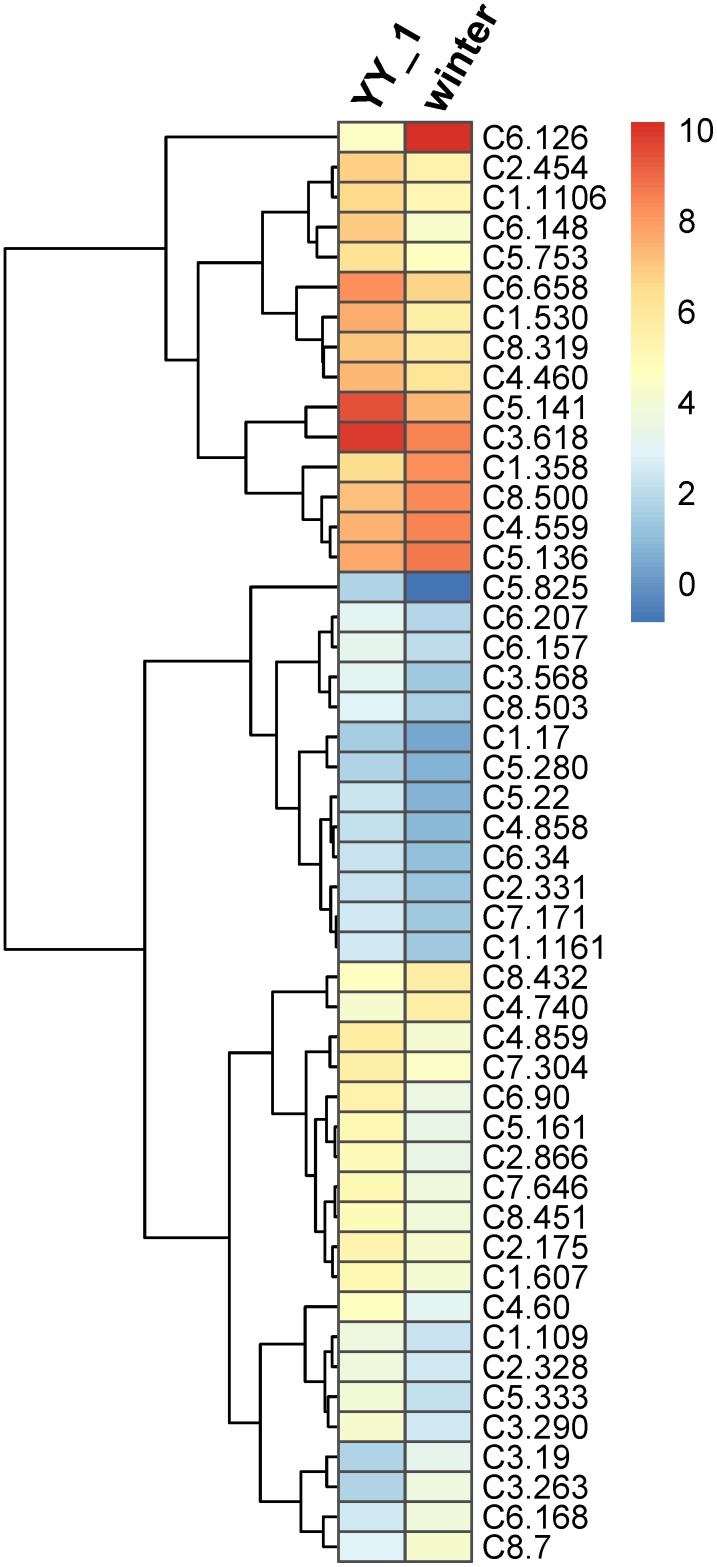
Expression profile of transcription factor genes with differential transcription levels in the *M. purpureus* winter strain compared to those in the YY-1 strain. The tree is a dendrogram of the cluster analysis obtained by the hclust method in R, which is a hierarchical partitioning of data that visually resembles a heat map and reflects the gene expression values in different strains.

Transcription factors play a critical role in the regulation of gene expression patterns that control the overall metabolism, including secondary metabolic pathways (Gutierrez et al., 2017; Wong and Matus, 2017). Therefore, the expression levels of transcription factor genes in *M. purpureus* during pigment and citrinin production were analyzed. Based on Pfam annotation, 179 genes were identified as transcription factor-encoding candidates, among which 80 showed significantly different expression levels in the *M. purpureus* winter strain compared to those in the YY-1 strain (**Supplementary Table S5**). Because of transcriptomic fluctuations, 48 transcription factor genes with more than a two-fold change were analyzed further. Of these genes, 37 showed decreased expression levels and 11 exhibited increased transcription levels in the winter strain (**Figure 5**) relative to the levels in the YY-1 strain, including the transcription activator C6.126. In the winter strain, the transcription levels of C5.141 and C3.618, which had respective RPKM values of 19 and 160, decreased by 4.3- and 2.7-fold, respectively, compared to those in the YY-1 strain.

These results indicated that the proteins encoded by *orf1*, *orf3*, and *ctnB* were essential but were not rate-limiting enzymes for citrinin production in *M. purpureus* and that the higher expression levels of these genes in the *M. purpureus* winter strain might result from the decreased expression levels of downstream citrinin synthesis pathway genes. In addition, some transcription factors, including C6.126, C5.141, and C3.618, may be involved in the regulation of citrinin biosynthesis. However, further investigations to elucidate the citrinin biosynthesis pathway in *M. purpureus* are necessary.

### A Comprehensive Analysis of the Biosynthesis Pathway of Pigment Hyper-Production

**FIGURE 6:**
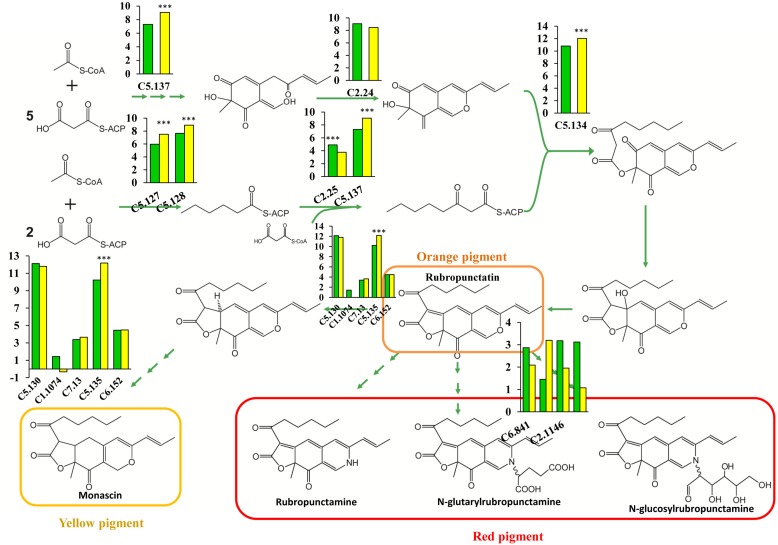
Schematic of pigment biosynthesis pathways in *M. purpureus*, including pathways for orange pigment (rubropunctatin), yellow pigment (monascin), red pigment (rubropunctamine), and water-soluble pigments. The histogram depicts the gene expression levels in the YY-1 (green) and winter (yellow) strains. The *y*-axis represents log2(RPKM), and ^∗∗∗^ denotes a *P*adj value < 0.001.

Pigment synthesis pathways in fungi have been attracting more attention, and great progress has been made in this area (Chiang et al., 2009; Mapari et al., 2010). PKS, which closely resembles animal fatty acid synthase (FAS) and is a large, multifunctional polypeptide composed of a set of catalytic domains, including β-ketoacyl synthase (KS), AT, and ACP domains, is the main participant in fungal pigment production (Thomas, 2001; Cox, 2007; Crawford and Townsend, 2010). Several novel enzymes involved in pigment synthesis have been found in *M. purpureus*, yet the importance of genes involved in pigment production has not been investigated, and the rate-limiting enzymes remain to be identified. In this study, we performed a comprehensive analysis of the pigment biosynthesis pathways in *M. purpureus*. PKS (C5.137) serves as a dual-functioning PKS and is responsible for the synthesis of 3-oxoacyl-thioester from acylthioester, showing an increased expression level with a 3.3-fold change in the winter strain (**Figure 6**). A similar reaction is catalyzed by the enzymes encoded by the FAS gene pair (*MpFasA* and *MpFasB*) in *M. azaphilone* (Balakrishnan et al., 2013). In addition, C5.134, a gene that encodes 3-O-acetyltransferase, exhibited high expression levels; its RPKM was 1798 and 4198 in the YY-1 and winter strains, respectively, suggesting the essential role of this gene in pigment production. Furthermore, the 2.33-fold upregulation of C5.134 in the winter strain suggested that C5.134 might be one of the rate-limiting enzymes. The expression levels of the FAS genes (C5.127 and C5.128) were significantly increased by 2.91- and 2.41-fold, respectively, in the winter strain; this result is similar to that in a previous study, which showed that pigment production is involved in fatty acid synthesis (Miazek et al., 2017). Intriguingly, the expression of oxidoreductase (C5.135), which is used to form yellow pigment from orange pigment, was upregulated by 3.9-fold, yet the genes encoding the enzymes involved in the catalytic reaction of rubropunctatin to red pigments showed low expression levels in the winter and parental strains, and the expression levels were not significantly different between the strains.

**FIGURE 7:**
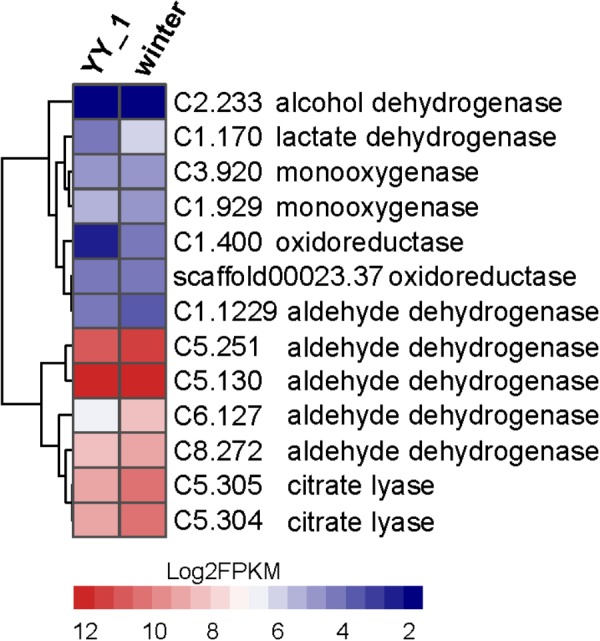
Expression levels of the genes involved in acetyl-CoA biosynthesis in the *M. purpureus* winter and YY-1 strains. The tree is a dendrogram of the cluster analysis obtained by the hclust method in *R*, which is a hierarchical partitioning of data that visually resembles a heat map and reflects the gene expression values in different strains.

The large transcriptional differences between the YY-1 and winter strains indicated the complex pigment synthesis pathway in *M. purpureus*. The most remarkable transcriptional changes are related to the acetyl-CoA metabolic network, pigment precursors and a crucial metabolite involved in both central carbon and energy metabolism. Several genes upregulated in the winter strain are involved in two potential pathways for acetyl-CoA biosynthesis (**Figure 7**), such as the genes encoding citrate lyase (C5.304 and C5.305) in the citrate pathway and the genes encoding oxidoreductase activity (C1.400 and C1.170) and aldehyde dehydrogenase (C5.251 and C6.127). Notably, the expression level of C6.127 was increased by 4.2-fold (from 84.06 to 353.68) in the *M. purpureus* winter strain. This observation was consistent with previous reports that fungi responding to carbon starvation stress on poor carbon sources can regulate the biosynthesis of secondary metabolism products (Jorgensen et al., 2009; Yang et al., 2015). In addition, when the KEGG function enrichment analysis of genes upregulated in the *M. purpureus* winter strain was performed, the genes involved in the glycolysis pathway, which provides acetyl-CoA for pigment production, were found to be enriched significantly (**Supplementary Table S6**). In accordance with these possibilities, the transcriptome data showed that many genes involved in the TCA cycle, such as citrate synthase (C4.56), and succinate dehydrogenase (C6.271 and C7.592), are downregulated in the winter strain; this downregulation does not favor the metabolic flux of acetyl-CoA to pigment.

The increased expression levels of key enzymes in the pentose phosphate pathway, 6-phosphogluconate dehydrogenase (C3.60) and glucose-6-phosphate dehydrogenase (C7.667), indicated enhanced NADPH regeneration. This observation suggested that secondary metabolite biosynthesis could be enhanced by an additional supply of NADPH. Oxidoreductase plays an essential role in secondary metabolite synthesis, especially pigment synthesis. In the winter strain, the genes (C5.133, C5.124, and C8.16) encoding oxidoreductase show significantly increased expression levels; these genes exhibited 4.0-, 5.65-, and 2.5-fold changes, respectively. This phenomenon is consistent with the results of previous reports that showed that increasing the expression level of MpigE, an ortholog of C5.133, promoted pigment biosynthesis in *Monascus ruber* M7 (Liu et al., 2014) and that azaH and tropB (orthologs of C5.124 and C8.16, respectively) were proposed to be involved in pyrone ring formation (Zabala et al., 2012; Balakrishnan et al., 2013). These results indicated that oxidoreductase-encoding genes may be candidates for the metabolic engineering of *Monascus* to aid the industrial use of secondary metabolites. Overall, the enhancement of central carbon metabolism, acetyl-CoA biosynthesis and NADPH regeneration led to a large increase in the production of pigments.

## Conclusion

This is the first report that a strain of *M. purpureus* with low citrinin secretion was obtained with random mutation with protoplast transformation and was analyzed by comparative transcriptomic techniques. We found that the expression level of the key gene involved in citrinin synthesis, *pksCT*, was low in both *M. purpureus* YY-1 and its mutant; this result suggested that citrinin synthesis might be catalyzed by isoenzymes that evolved in the *M. purpureus* YY-1 strain. Furthermore, the expression analysis of the transcription factor genes can promote the study of networks regulating the citrinin synthesis pathway in *M. purpureus.* Moreover, the production of the three pigments (red, yellow, and orange) in the winter mutant during the whole fermentation process was significantly increased. Transcriptome analysis showed that central carbon metabolism, acetyl-CoA biosynthesis and NADPH regeneration were enhanced in the winter strain; these pathways can contribute to the high yield of pigments. This study improves our understanding of pigment and citrinin production in *M. purpureus* and benefits the application of *M. purpureus* in the production of food and pharmaceuticals.

## Author Contributions

SW and X-JD designed the project. BL carried out the experiments. BL, X-JD, PL, and C-CS participated in the data analysis and wrote the manuscript. All authors read and approved the final manuscript.

## Conflict of Interest Statement

The authors declare that the research was conducted in the absence of any commercial or financial relationships that could be construed as a potential conflict of interest.

## Footnotes

1 http://www.bioinformatics.babraham.ac.uk/projects/fastqc/

## Supplementary Material

FIGURE S1 Correlation comparison between the qRT-PCR results and the RNA-Seq data. The relative expression levels of the selected genes were normalized to their expression in wild-type strains. The Spearman correlation coefficients and P values are shown in the top left of each figure. For the qRT-PCR results (horizontal coordinate), the values represent the means of triplicates.

TABLE S1 The profiles of RNA-seq reads mapped to the genome of *Monascus purpureus* and the differential expression analysis.

TABLE S2 Gene ontology analysis of 1377 genes with up-regulated expression levels in *M. purpureus* winter. *p*-values highlighted in yellow indicate functional classifications that were significantly enriched.

TABLE S3 Gene ontology analysis of 1418 genes with down-regulated expression levels in *M. purpureus* winter. p-values highlighted in yellow indicate functional classifications that were significantly enriched.

TABLE S4 Genes involved in carbohydrate degradation was significantly decreased in *M. purpureus* winter, belonging to the cluster of carbohydrate metabolic process.

TABLE S5 Expression profile of transcription factor genes with differential transcriptional levels and more than two-fold charges in *M. purpureus* winter, compared to that in the YY-1 strain.

TABLE S6 The analysis of KEGG function enrichment of up-regulated genes in M. purpureus winter in *M. purpureus* winter. *p*-values highlighted in yellow indicate functional classifications that were significantly enriched.

TABLE S7 RT-PCR Primers used in this study.
