# Supplementary figures and images for: Investigation of Citrinin and Pigment Biosynthesis Mechanisms in Monascus purpureus by Transcriptomic Analysis
Source: Front Microbiol. 2018 Jun 28;9:1374. doi: 10.3389/fmicb.2018.01374 (PMC6031731; doi:10.3389/fmicb.2018.01374)

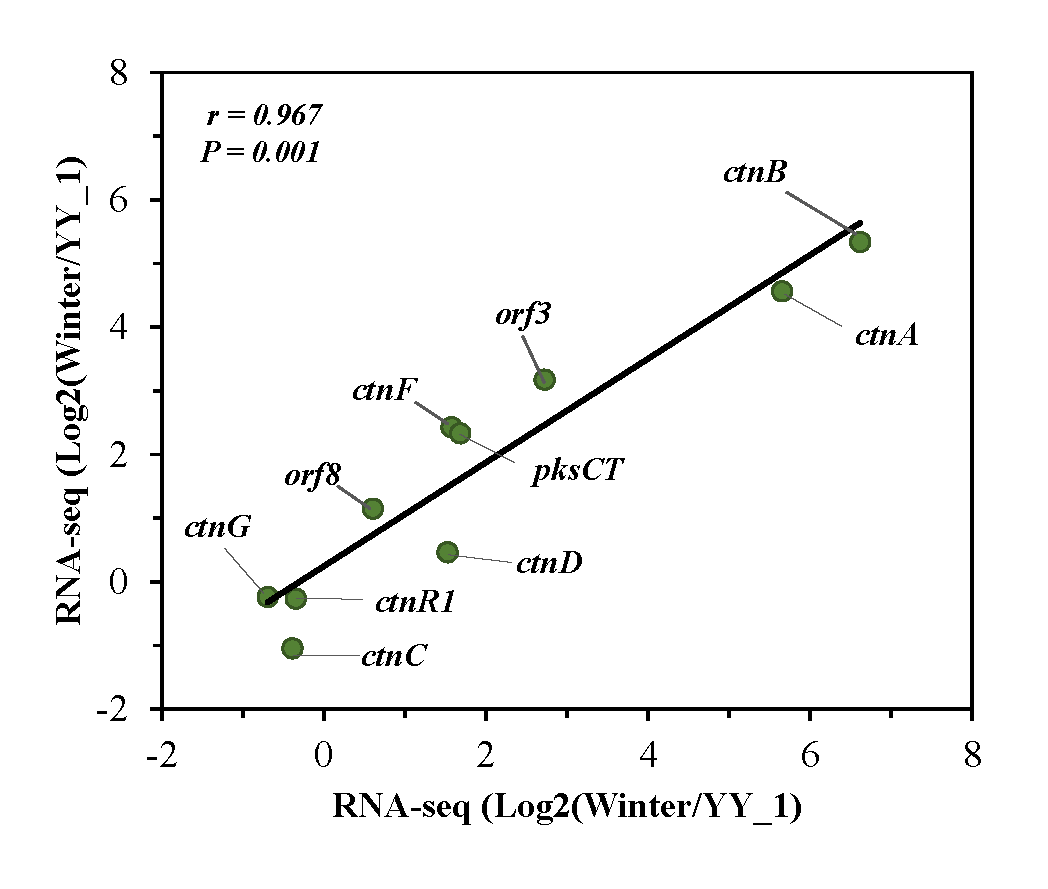

Supplement: FIGURE S1 — Correlation comparison between the qRT-PCR results and the RNA-Seq data. The relative expression levels of the selected genes were normalized to their expression in wild-type strains. The Spearman correlation coefficients and P values are shown in the top left of each figure. For the qRT-PCR results (horizontal coordinate), the values represent the means of triplicates. [file Image_1.TIF]
